# Rectosigmoid-Junction Squamous Cell Carcinoma With pMMR/MSS Achieved a Partial Response Following PD-1 Blockade Combined With Chemotherapy: A Case Report

**DOI:** 10.3389/fonc.2021.596342

**Published:** 2021-05-25

**Authors:** Yanxin He, Lunqing Wang, Xiao Li, Tongsong Zhang, Tingting Song, Junling Zhang, Yangyang Yu, Shiqing Chen, Haiping Song

**Affiliations:** ^1^ Department of Internal Medicine-Oncology, Qingdao Tumor Hospital, The Second Affiliated Hospital of Medical College of Qingdao University, Qingdao, China; ^2^ Department of Thoracic Surgery, Qingdao Municipal Hospital, Qingdao, China; ^3^ The Medical Department, 3D Medicines Inc., Shanghai, China

**Keywords:** rectosigmoid-junction squamous cell carcinoma, proficient mismatch repair/microsatellite stability, programmed death ligand-1 blockade, programmed death ligand-1 expression, tumor mutation burden

## Abstract

Colorectal squamous cell carcinoma (SCC) is extremely rare and associated with a poor prognosis. And the pMMR/MSS colorectal cancer is related to a limited response to programmed death ligand-1 (PD-1) blockade monotherapy. However, the clinical activity of PD-1 blockade monotherapy or combination therapy in colorectal SCC is unknown. One patient with rectosigmoid-junction SCC was treated with PD-1 blockade combined with chemotherapy. After 3 months of PD-1 blockade and chemotherapy, the computed tomography imaging showed that this patient achieved a partial response. The next generation sequencing and immunohistochemistry analysis showed that the patient had tumors with proficient mismatch repair (pMMR) and microsatellite stability (MSS), strong PD-L1 expression, and tumor mutational burden-high (TMB-High), respectively. This case suggests that PD-1 blockade combined with chemotherapy might be an effective therapy for colorectal SCC with pMMR/MSS status. Moreover, the PD-L1 expression and TMB might be the potential predictors of PD-1 blockade response for colorectal SCC patients.

## Introduction

Primary squamous cell carcinoma (SCC) of the colorectum is extremely rare, reportedly comprising 0.25% to 0.85% of all colorectal carcinomas. Colorectal SCC generally presents at an advanced stage and is associated with a poor prognosis (1). Comparing with adenocarcinomas, survival in colorectal SCC is poorer (2). Chemoradiation has been used as a primary treatment for colorectal SCC, with no universally accepted regimen. However, given to the poor outcome, more effective and standard treatments are urgently needed.

Blocking programmed death-1 (PD-1)/programmed death ligand-1 (PD-L1) therapy has exhibited promising efficacy in some colorectal cancer (CRC). Studies have observed that CRC patients with deficient mismatch repair (dMMR) or microsatellite instability- high (MSI-H) exhibited higher response rates to PD-1 blockade monotherapy (3). The National Comprehensive Cancer Network clinical practice guidelines in colon/rectum cancer Version 4.2020 recognizes either Nivolumab, Nivolumab+Ipilimumab, or Pembrolizumab as acceptable standard of care treatment options for patients with dMMR/MSI-H metastatic CRC that have progressed after first-line chemotherapy. Unfortunately, only about 3% to 6% of advanced staged colorectal patients exhibited dMMR or MSI-H characters (4). Summarizing the previous clinical trials, PD-1/PD-L1 monotherapy did not show promising results in proficient mismatch repair (pMMR) or microsatellite stability (MSS) tumors (5). Multiple ongoing studies are exploring the combination modality, including 5FU-based regimens, radiotherapy, EGFR inhibitors, VEGF inhibitors and so on (6). Hence, it is worth and important to continue to investigate the role of immunotherapy in pMMR/MSS CRC.

Besides MSI status, tumor mutation burden (TMB) and PD-L1 expression are the most promising biomarkers of immunotherapy in CRC. Recently, the Food and Drug Administration (FDA) has approved Pembrolizumab for the treatment of adult and pediatric patients with unresectable or metastatic solid tumors with tissue tumor mutational burden-high (TMB-High, ≥10 mutations/mb). And PD-L1 expression has been extensively evaluated as a predictive biomarker of anti-PD-1 therapy in some tumor types, such as non-small-cell lung carcinoma, gastric cancer, and oesophageal cancer (7). In MSI-high CRC, PD-L1 expression is observed to act as a regulatory factor for immune response (8). Consequently, the TMB and PD-L1 expression has been associated with a response to immunotherapy in CRC patients. Here, we reported one rectal SCC patient that was successfully treated with PD-1 blocker plus chemotherapy, who showed high PD-L1 expression and TMB-High with a pMMR/MSS status.

## Case Presentation

**Figure 1:**
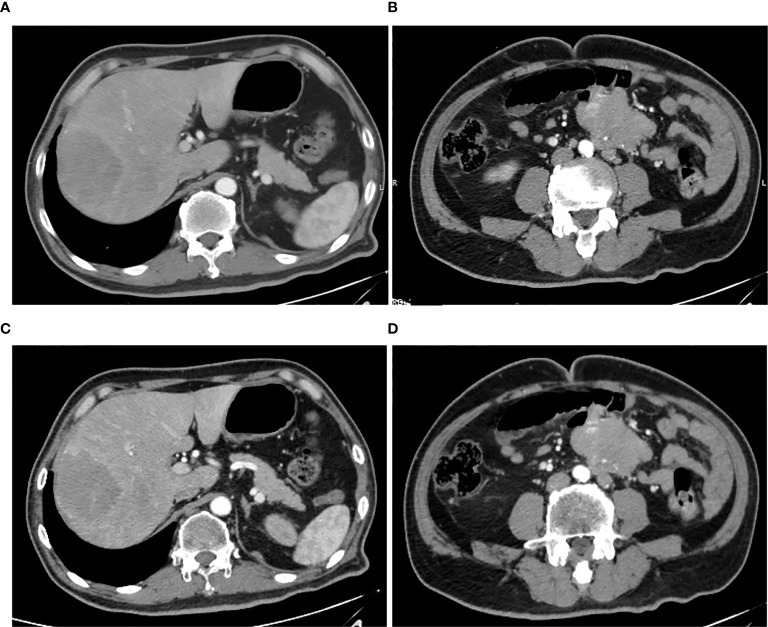
Pretreatment CT images. **(A, B)** CT arterial and portal phase images of liver metastasis with a size of 70 mm × 61 mm, respectively. **(C, D)** CT arterial and portal phase images of abdominal & pelvic cavity metastasis with a size of 76 mm × 50 mm, respectively.

**Figure 2:**
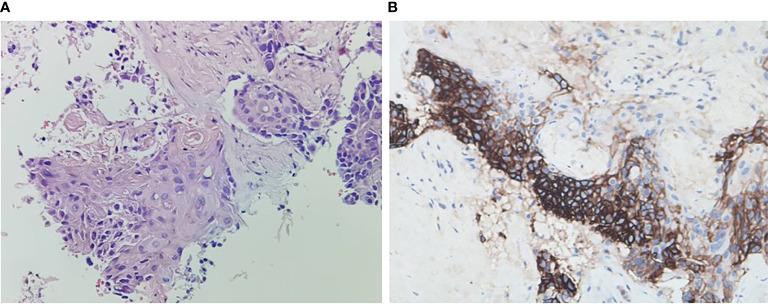
Histopathology of rectosigmoid-junction SCC cancer and immunohistochemistry. **(A)** H & E stain, original magnification ×200. **(B)** PD-L1 immunohistochemistry (antibody Dako 22C3), original magnification ×200.

**Figure 3:**
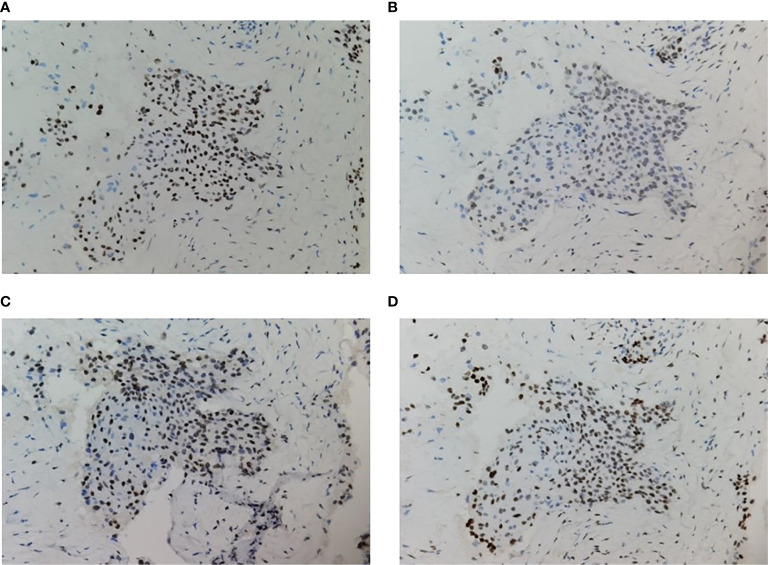
Immunohistochemistry of MMR proteins. The tumor cells preserved expression of MLH1 **(A)**, MSH2 **(B)**, PMS2 **(C)**, and MSH6 **(D)**; original magnification ×200.

**Figure 4:**
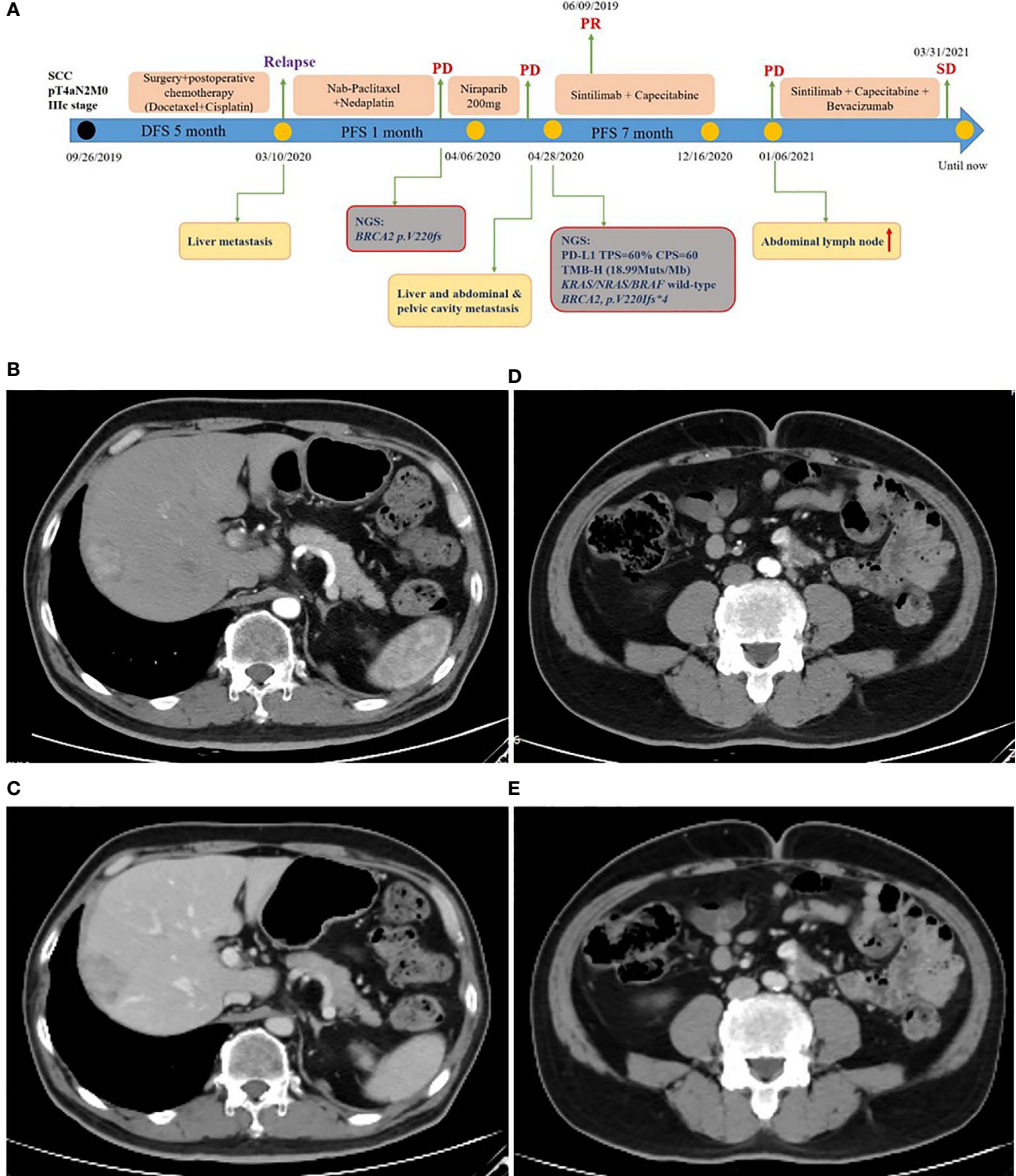
Treatment of rectosigmoid-junction SCC cancer. **(A)** Timeline of the clinical course in this patient. **(B, C)** CT scan showed a partial response in liver metastasis (40 mm × 27 mm) from arterial and portal phase images. **(D, E)** CT scan showed a partial response in abdominal & pelvic cavity metastasis (32 mm × 18 mm) from arterial and portal phase images.

A 59-year-old man experienced a surgery of lung cancer in 2016. He was diagnosed with primary rectosigmoid-junction SCC (pT4aN2M0 IIIc, tumor size: 10 cm×9 cm) and underwent radical resection in September, 2019. After surgery, he was treated with the chemotherapy (Docetaxel+Cisplatin). In March 2020, liver metastasis was found and he was treated with the chemotherapy (Nab-Paclitaxel + Nedaplatin). During the chemotherapy, the patient showed a progressive disease. A genetic testing was used to the surgery tissue and a *BRCA2 p.V220fs* mutation was found. According to the mutation of *BRAC2*, the treatment was changed to Niraparib. But the tumor continued to progress. In April 2020, this patient was admitted to our hospital. The computed tomography (CT) revealed metastasis in the liver and abdominal & pelvic cavity (**Figure 1**). Biopsy was performed, and the pathology of the specimen revealed SCC (**Figure 2A**). Immunohistochemistry (IHC) showed a strong positive expression of PD-L1 in tumor cells (**Figure 2B**). Next generation sequencing (NGS) of biopsy tissue was performed on Illumina HiSeq sequencer (Illumina, San Diego, CA) with a median unique exon coverage depth of 800× in a College of American Pathologists (CAP) and Clinical Laboratory Improvement Amendments (CLIA) certified laboratory (3D Medicine Inc., Shanghai, China) (9). The NGS results showed that the *KRAS*/*NRAS*/*BRAF* genes were wild-type. And this patient harbored one germline mutation in *BRCA2*, p.V220Ifs*4. The TMB-H (18.99 Muts/Mb) and MSS were identified. No SNVs were detected in *MLH1*, *MSH2*, *MSH6* and *PMS2*. The IHC method was used to confirm that the patient had pMMR rectosigmoid-junction SCC cancer (**Figures 3A–D**). In April 28, 2020, the patient was received Sintilimab, a PD-1 antibody (200 mg on D1, every 3 weeks) (**Figure 4A**). And on May 12, 2020, the chemotherapy was added (Capecitabine 1.5 g, twice a day on D1-D14, every 3 weeks). About three months later, a partial response was confirmed in the CT scan (**Figures 4B–E**) and lasted until January 6, 2021. In January 2021, lymph node enlargement was found and he was treated with the Sintilimab plus Capecitabine and Bevacizumab (200 mg on D1). Currently, at the end of Mar. 2021, the patient was continuing the immunotherapy combined with chemotherapy and a stable response was confirmed.

## Discussion

This is the first report demonstrating one rectosigmoid-junction SCC patient with pMMR/MSS achieved a partial response after PD-1 blockade combined with chemotherapy. Meanwhile, the patient had shown high PD-L1 expression and TMB-High.

The KEYNOTE series studies had reported that an excellent response rate of Pembrolizumab in several advanced PD-L1-positive malignancies (10). However, PD-L1 expression had not yet been approved as predictive biomarker for PD-1 blockade therapy in CRC. A gastrointestinal cancer statistical study reported that colorectal adenocarcinoma always harbored low PD-L1 expression and MSI-H, but esophageal SCC always harbored high PD-L1 expression and MSS (11). This might indicate that there was a difference of PD-L1 expression between SCC and adenocarcinoma in gastrointestinal cancers. However, data about PD-L1 expression level in colorectal SCC patients are limit. Our case report indicates that high PD-L1 expression might be associated with an increased response to PD-1 blockade treatment in colorectal SCC patients. Thus, for the colorectal SCC patients, especially the ones with MSS status, it might be valuable to evaluate PD-L1 expression in anti-PD-1 treatment decision.

Focusing on TMB, the FDA granted approval to Pembrolizumab for the treatment of patients with TMB-High solid tumor. Thus, for colorectal SCC patients, TMB had become the next predictive biomarker for PD-1 blockade treatment. The TMB of our case was 18.99 mutations/Mb, defining TMB-High and predicting a good efficacy of anti-PD-1 therapy. Studies had shown that TMB-High had a strong association with MSI-H in most gastrointestinal tumors, indicating that MSI-H was the main cause of TMB-high. But in some gastrointestinal SCC with MSS status, such as anal and esophageal cancers, could be shown as TMB-High (11). Therefore, TMB-High might be a more appropriate biomarker for anti-PD-1 immunotherapy in colorectal SCC patients.

Anti-PD-1 monotherapy provided limited clinical benefit in CRC patients with pMMR/MSS tumors. Recently, more studies had been actively exploring the efficacy of immunotherapy combination treatments to increase immune response. In a phase 2 study, patients with advanced refractory CRC received the combined immune checkpoint inhibition (Durvalumab and Tremelimumab), which resulted in significant improvement in median OS. Patients who were MSS with plasma TMB ≥ 28 mutations/Mb had the greatest OS benefit (12). PD-1 blockade combined with anti-angiogenesis therapy also showed promising efficacy in MSS CRC. In the REGONIVO trial, ORR was 33% in advanced CRC patients with MSS status that received Nivolumab and Regorafenib (13). In our case, the patient was treated with PD-1 blocker combined with chemotherapy and achieved significant clinical benefit. Until the case was written, the patient had been continuing the combined therapy with excellent performance status and quality of life.

The colorectal SCC is a quite rare tumor. There are no large cohort studies and standard clinical treatment guidelines. Our case report indicates that anti-PD-1 therapy with chemotherapy may have a promising anti-tumor activity in rectosigmoid-junction SCC. TMB and PD-L1 expression might be the potential predictors for colorectal SCC patients who would benefit from PD-1 blockade. However, further clinical studies with larger samples are required to validate the clinical activity of PD-1 blockade in colorectal SCC patients and relevant predictive biomarkers.

## Data Availability Statement

The original contributions presented in the study are included in the article/supplementary material. Further inquiries can be directed to the corresponding author.

## Ethics Statement

Written informed consent was obtained from the individual(s) for the publication of any potentially identifiable images or data included in this article.

## Author Contributions

YH, LW, and HS conceived the experiments and wrote the paper. XL, TZ and TS helped with experiments. YY, SC, and JZ analyzed the data. All authors contributed to the article and approved the submitted version.

## Funding

This work was supported by grants from Qingdao Annual Industrial Cultivation Plan for Science and Technology (no. 18-6-1-98-nsh), Qingdao Expert Workstation (2018 no. 165), Beijing Xisike-BMS Clinical Oncology Research Foundation (Y-BMS2019-035).

## Conflict of Interest

JZ, YY, and SC were employed by the company 3D Medicines Inc., Shanghai, China.

The remaining authors declare that the research was conducted in the absence of any commercial or financial relationships that could be construed as a potential conflict of interest.
